# HIV-1 subverts the complement system in semen to enhance viral transmission

**DOI:** 10.1038/s41385-021-00376-9

**Published:** 2021-02-10

**Authors:** Bernadien M. Nijmeijer, Marta Bermejo-Jambrina, Tanja M. Kaptein, Carla M. S. Ribeiro, Doris Wilflingseder, Teunis B. H. Geijtenbeek

**Affiliations:** 1 Department of Experimental Immunology, Amsterdam Infection and Immunity Institute, Amsterdam University Medical Centers, University of Amsterdam, Amsterdam, the Netherlands; 2 Institute of Hygiene and Medical Microbiology, Medical University of Innsbruck, Innsbruck, Austria

## Abstract

Semen is important in determining HIV-1 susceptibility but it is unclear how it affects virus transmission during sexual contact. Mucosal Langerhans cells (LCs) are the first immune cells to encounter HIV-1 during sexual contact and have a barrier function as LCs are restrictive to HIV-1. As semen from people living with HIV-1 contains complement-opsonized HIV-1, we investigated the effect of complement on HIV-1 dissemination by human LCs in vitro and ex vivo. Notably, pre-treatment of HIV-1 with semen enhanced LC infection compared to untreated HIV-1 in the ex vivo explant model. Infection of LCs and transmission to target cells by opsonized HIV-1 was efficiently inhibited by blocking complement receptors CR3 and CR4. Complement opsonization of HIV-1 enhanced uptake, fusion, and integration by LCs leading to an increased transmission of HIV-1 to target cells. However, in the absence of both CR3 and CR4, C-type lectin receptor langerin was able to restrict infection of complement-opsonized HIV-1. These data suggest that complement enhances HIV-1 infection of LCs by binding CR3 and CR4, thereby bypassing langerin and changing the restrictive nature of LCs into virus-disseminating cells. Targeting complement factors might be effective in preventing HIV-1 transmission.

## Introduction

Even though the number of new HIV-1 infections globally continues to decline, with 1.7 million new infection in 2018^1^, the pandemic is still a major health burden. Currently there is no curative treatment or vaccine to prevent HIV-1 infection. Sexual transmission of HIV-1 is the most common route of infection^2,3^. HIV-1 susceptibility is affected by different host factors^4^ such as the physical barrier of the mucosa^5,6^, or immune activation by genital inflammation established by other sexual transmitted infections^7–9^. But also, donor factors such as composition of semen can modulate susceptibility. Semen contains proteins including semen-derived enhancer of viral infection (SEVI) that enhance viral infection^10–12^. Semen also contains complement (C) factors^12^. As the HIV-1 glycoprotein gp41 expresses a C-activating domain, the interaction of HIV-1 with complement factors results in iC3b-opsonization of HIV-1^13–15^. However, HIV-1 prevents complement dependent lysis by acquiring host cell-derived proteins during the budding process^16^. Interestingly, several studies suggest that complement-opsonized HIV-1 differently interacts with immune cells, modulating immunity, and enhancing infection^10,12^. However, little is known about the role of complement in semen in HIV-1 transmission across mucosal tissues. Langerhans cells (LCs), based on their function, are commonly classified as a subset of dendritic cells (DCs) that are present at the mucosal epithelia of the vagina, foreskin, and anal tissues^17–19^ and are therefore the first immune cells to encounter HIV-1 during sexual contact^20,21^. In steady-state conditions LCs form a protective barrier against HIV-1 infection by capture of HIV-1 via C-type lectin receptor langerin (CD207) and subsequent degradation via TRIM5α-mediated autophagy^20,21^. Notably, activation of LCs by inflammation or genital co-infections breaches the protective function of LCs, leading to LC infection and promoting HIV-1 transmission to T cells^7,19,22,23^. Here we have investigated the role of complement present in semen in HIV-1 susceptibility using LCs in an ex vivo human tissue infection model. Our data strongly suggest that complement opsonization of HIV-1 in human seminal fluid (SF) abrogates LC restriction leading to infection of LCs ex vivo and subsequent viral transmission. Infection of LCs was dependent on complement receptors 3 (CR3; CD11b/CD18) and complement receptor 4 (CR4; CD11c/CD18), underscoring the role of the complement system in SF as an important enhancer of HIV-1 susceptibility. Complement-opsonized HIV-1 capture by CR3 and CR4 prevented langerin dependent degradation and enhanced HIV-1 fusion, integration, and transcription. Thus, HIV-1 subverts complement present in SF to enhance HIV-1 susceptibility. Novel therapeutical interventions targeting CR3 and CR4 could be considered to counteract cell mediated dissemination of HIV-1.

## Results

### Human semen enhances HIV-1 transmission by Langerhans cells ex vivo

**Fig. 1:**
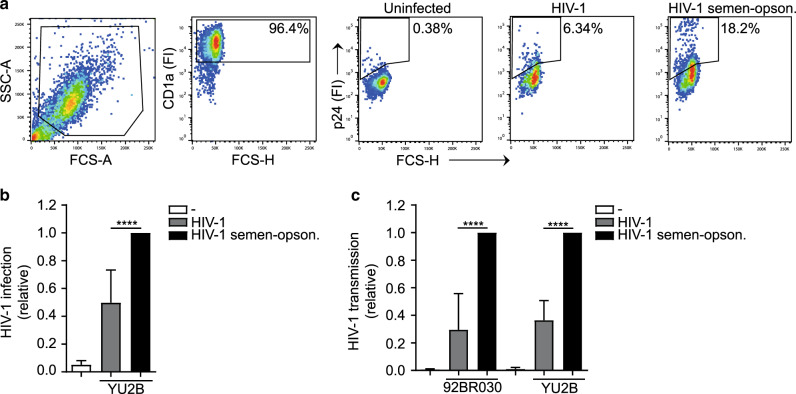
Human seminal fluid enhances HIV-1 transmission by Langerhans cells ex vivo. **a**–**c** Epidermal sheets were exposed to HIV-1 (YU2B or 92BR030) or semen-opsonized HIV-1 (YU2B or 92BR030). **a**, **b** Harvested, stained with antibodies against CD1a, CD207, and p24 and analyzed by flow cytometry. **a** Representative flow cytometry plots of one donor. The percentage of CD1a positive cells is 96.4% and depicted in the upper-right corner of the dot plot, the percentage of infected cells is depicted in the dot plot. **b** Levels of infection were calculated relative to the percentage of infection and set at 1 in cells exposed to semen-opsonized HIV-1. **c** Harvested, extensively washed and co-cultured with U87 cells. Transmission was determined by antibody staining against p24 and analyzed by flow cytometry. Levels of transmission were calculated relative to the percentage of infection in U87 cells and set at 1 in cells exposed to semen-opsonized HIV-1. Error bars are the mean ± SD of **b** *n* = 4 donors (YU2B) measured in duplicate. With absolute values of p24% for UI: 0.99; 0.31; 0.65; 0.33, for HIV-1: 2.51; 20.45; 7.41; 2.99, and for semen-opsonized HIV-1: 15.55; 41.7; 11.05; 4.47. ****p < 0.0001 by ordinary one-way ANOVA, Tukey post hoc test (multiple comparisons). **c** *n* = 3 donors (92BR030) and *n* = 3 donors (YU2B) measured in duplicate. With absolute values of p24% (92BR030) for UI: 0.02; 0.04; 0.07, for HIV-1: 1.49; 2.35; 0.58, and for semen-opsonized HIV-1: 2.53; 11.81; 5.85. With absolute values of p24% (YU2B) for UI: 0.18; 0.05; 0.03, for HIV-1: 2.07; 2.38; 8.72, and for semen-opsonized HIV-1: 7.89; 8.34; 16.0. ****p < 0.0001 by two-way ANOVA, Tukey post hoc test (multiple comparisons).

As semen has been shown to affect infection by HIV-1^10,12^, we investigated the role of semen in HIV-1 transmission by LCs using the ex vivo tissue model. Human skin tissue was exposed to 100 ul/sheet and 500 ng p24/ml of HIV-1 92BR030 primary isolate or HIV-1 YU2B molecular clone, respectively, and after several days culture, migrated LCs were analyzed for infection. High concentrations of HIV-1 led to infection of LCs ex vivo but, notably, pre-treatment of HIV-1 with SF significantly increased HIV-1 infection of LCs, independently of the viral strain (Fig. 1a, b). Moreover, SF treatment of HIV-1 strains YU2B and 92BR030 enhanced HIV-1 transmission by LCs to highly susceptible target cells in the ex vivo tissue transmission model (Fig. 1c). Semen treatment of HIV-1 had no effect on target cell infection (Supplementary Fig. S1). These data strongly suggest that semen enhances LC infection and transmission by HIV-1 ex vivo.

### Complement receptor 3 and 4 on Langerhans cells capture complement-opsonized HIV-1

**Fig. 2:**
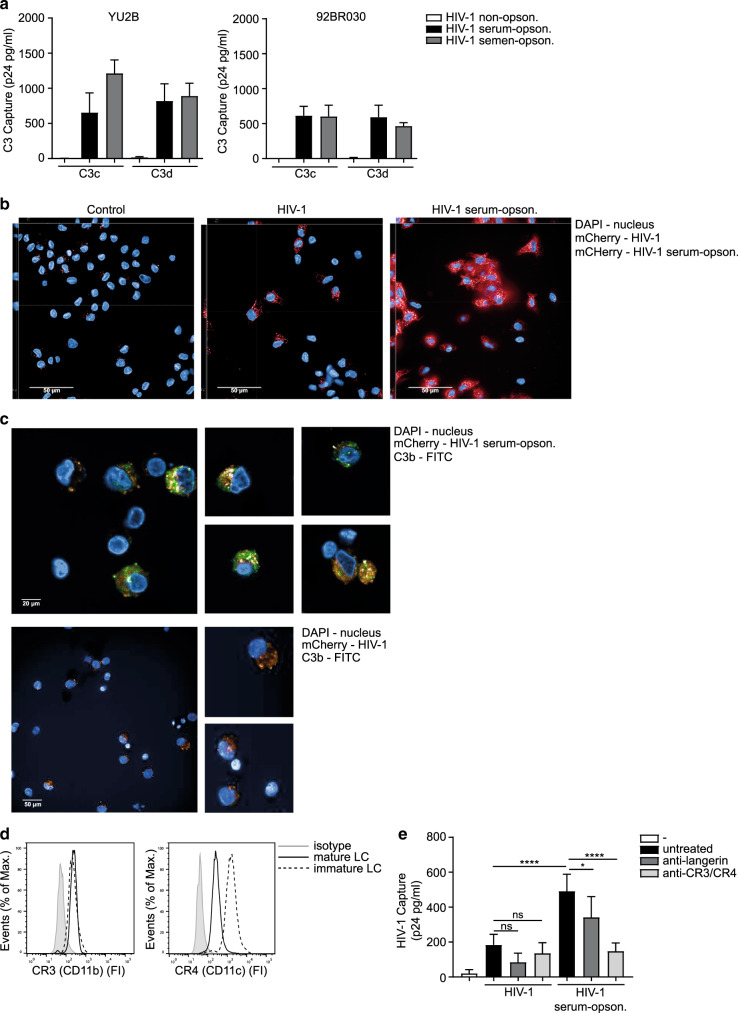
Complement receptor 3 and 4 on Langerhans cells capture complement-opsonized HIV-1. **a** HIV-1 opsonization patterns as determined by VCA using anti-human C3c (recognizing C3b, iC3b) and C3d. Binding was quantified by p24 ELISA. **b** Confocal microscopy analysis of mature LCs exposed to YU2B/mCherry (250 ng p24/ml) or serum-opsonized YU2B/mCherry (250 ng p24/ml), permeabilized and stained with antibodies against DAPI. HIV-1 and serum-opsonized HIV-1 shown in red (mCherry), nucleus shown in blue (DAPI). Bars = 50 µm. Left and upper part of each picture show xyz-stack side view (scale bar = 5 µm). **c** Confocal microscopy analysis (original, 63xWATER lens) of mature LCs exposed to serum-opsonized YU2B/mCherry (350 ng p24/ml) or YU2B/mCherry (350 ng p24/ml), permeabilized and stained with antibodies against C3b and DAPI. HIV-1 shown in orange (mCherry), complement shown in green (C3b-FITC), nucleus shown in blue (DAPI). **d** Immature and mature LCs express CR3 (CD11b) and CR4 (CD11c) on the cell surface as determined by flow cytometry. One representative donor out of 3 is depicted. **e** Mature LCs were pre-exposed to anti-langerin or anti-CR3/CR4 Ab for 2 h and subsequently exposed to HIV-1 (JRCSF) or serum-opsonized HIV-1 (JRCSF) for 6 h, washed, lysed, and quantified by p24 ELISA. Error bars are the mean ± SD of **a** *n* = 3 donors (YU2B) *n* = 3 donors (BR92030) measured in duplicate. **e** *n* = 3 donors measured in duplicate. ns = not significant (*p* = 0.0975) (*p* = 0.9750), *p < 0.05, ****p < 0.0001 by two-way ANOVA, Tukey post hoc test (multiple comparisons).

Next, we investigated whether semen-enhanced transmission is dependent on complement opsonization by determining covalent deposition of C3 fragments, C3c (recognizing C3b and iC3b), and C3d on HIV-1 virions^15^. Treatment of YU2B, 92BR030, and JRCSF with human SF as well as pooled normal human serum (NHS) led to the deposition of C3c and C3d fragments on HIV-1, while no C3 deposition was measured with non-opsonized HIV-1 (Fig. 2a and Supplementary Fig. S2). These data strongly suggest that HIV-1 in semen is opsonized by complement. As we did not observe differences in complement between serum and semen, we used NHS to complement opsonize HIV-1. We further investigated whether complement-opsonized HIV-1 is internalized into LCs. LCs were exposed to complement-opsonized HIV-1 and internalization was analyzed by confocal microscopy. LCs expressed high levels of langerin and CD1a (Supplementary Fig. S3). Mature LCs capture HIV-1, which was enhanced when HIV-1 was complement-opsonized (Fig. 2b and Supplementary Fig. S4). Complement-opsonized HIV-1 co-localized with C3b protein within mature LCs, whereas no C3b protein was detected in HIV-1-exposed cells (Fig. 2c). These data suggest that LCs capture and internalize complement-opsonized HIV-1. As both immature and mature LCs expressed the CR3 (CD11b) and CR4 (CD11c) (Fig. 2d), we investigated their function in HIV-1 capture using blocking antibodies against CR3, CR4, and langerin. As shown before, LCs captured HIV-1 via langerin (Fig. 2e)^20^. Uptake of complement-opsonized HIV-1 by LCs was significantly increased compared to untreated HIV-1 (Fig. 2e). Antibodies against CR3 and CR4 blocked the uptake of opsonized HIV-1 (Fig. 2e). Blocking langerin reduced HIV-1 capture but did not significantly reduce capture of complement-opsonized HIV-1 (Fig. 2e). These results strongly suggest that complement opsonization increases HIV-1 binding and internalization into LCs via CR3 and CR4.

### Complement opsonization enhances HIV-1 fusion, integration, and infection of mature Langerhans cells

**Fig. 3:**
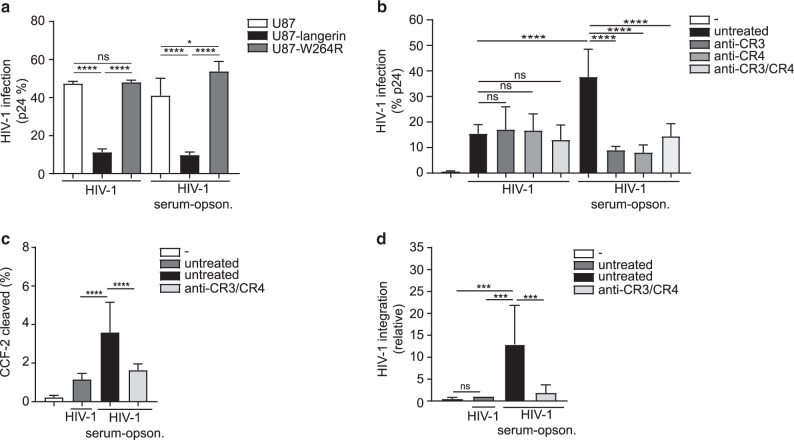
Complement opsonization enhances HIV-1 fusion, integration, and infection of mature Langerhans cells. **a** U87, U87-langerin or U87-W264R cell lines were infected with HIV-1 (JRCSF) or serum-opsonized HIV-1 (JRCSF), stained with an antibody against p24 and analyzed by flow cytometry. **b** Mature LCs were incubated with anti-CR3/CR4 antibody and exposed to HIV-1 (JRCSF) or serum-opsonized HIV-1 (JRCSF) stained with antibodies against CD1a and p24 and analyzed by flow cytometry. **c** Mature LCs were incubated with anti-CR3/CR4 antibody and subsequently exposed to non-opsonized or serum-opsonized HIV-1 containing the chimeric protein β–lactamase-Vpr (Blam-Vpr). CCF2-AM substrate cleavage was analyzed by flow cytometry with CD1a as a marker for LCs. **d** Mature LCs were incubated with anti-CR3/CR4 antibody and subsequently exposed to HIV-1 (Bal) or serum-opsonized HIV-1 (Bal). LC-integrated HIV-1 DNA was determined by a two-step Alu-PCR. Error bars are the mean ± SD of **a** *n* = 3 experiments measured in duplo, **b** *n* = 4 donors measured in duplicate, **c** *n* = 3 donors measured in duplicate, **d** *n* = 4 donors measured in duplicate. **a** ns = not significant (*p* = 0.9796), *p < 0.05, ****p < 0.0001 by two-way ANOVA, Tukey post hoc test (multiple comparisons). **b** ns = not significant (*p* > 0.9999), (*p* > 0.9999), (*p* = 0.9981), ****p < 0.0001 by two-way ANOVA, Tukey post hoc test (multiple comparisons). **c** ****p < 0.0001 by ordinary one-way ANOVA, Tukey post hoc test (multiple comparisons). **d** ns = not significant (*p* = 0.9962) ***p < 0.001 by ordinary one-way ANOVA, Tukey post hoc test (multiple comparisons).

Endogenous langerin on LCs as well as ectopic expression of langerin on HIV-1 susceptible U87 cell line restricts HIV-1 infection via TRIM5α-dependent autophagy^21^. Therefore, we investigated whether langerin is able to restrict complement-opsonized HIV-1 infection in the absence of CR3 and CR4 as U87 cells did not express both CR3 and CR4 (Supplementary Fig. S5). Ectopic expression of langerin abrogated infection of U87 cells with complement-opsonized HIV-1 to similar extent as untreated HIV-1 (Fig. 3a) in contrast to the langerin W264R mutant that is unable to bind HIV-1 (Fig. 3a). These data suggest that langerin is able to restrict complement-opsonized HIV-1 in the absence of CR3 and CR4. Hence, we examine the function of CR3 and CR4 in infection of LCs with complement-opsonized HIV-1. JRCSF HIV-1 was exposed to either medium or NHS and directly used for infection. Complement-opsonized HIV-1 infected LCs to a significantly higher extent than non-opsonized HIV-1, which was inhibited by antibodies against CR3 and/or CR4 (Fig. 3b and Supplementary Figs. S6 and S7). In contrast, antibodies against CR3 and CR4 did not affect non-opsonized HIV-1 infection, strongly suggesting that neither CR3 nor CR4 interacts with other proteins on the virus envelope (Fig. 3b). Heat-inactivation of complement resulted in a decrease of LC infection by JRCSF and YU2B to levels similar as observed for non-opsonized HIV-1 (Supplementary Fig. S8). Next, we investigated the pathways involved in increased infection by complement-opsonized HIV-1. Interestingly, complement opsonization strongly enhanced HIV-1 fusion as well as integration in a CR3 and CR4 dependent manner (Fig. 3c, d). Thus, these data strongly suggest that even though langerin is able to degrade complement-opsonized HIV-1, CR3 and CR4 enhance complement-opsonized HIV-1 infection of LCs by increasing fusion and subsequent integration.

### Complement opsonization enhances HIV-1 transmission by Langerhans cells via complement receptor 3 and 4

**Fig. 4:**
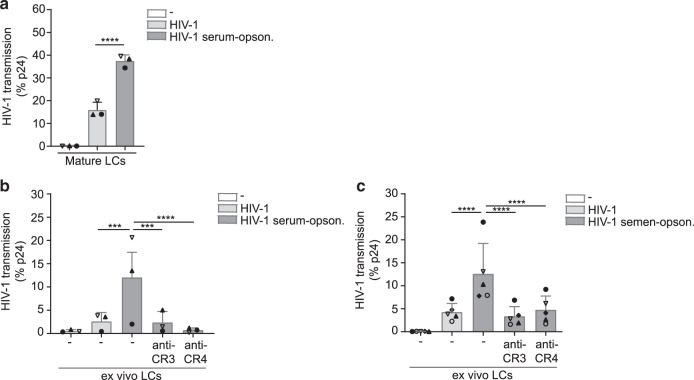
Complement opsonization enhances HIV-1 transmission by Langerhans cells via complement receptor 3 and 4. **a** Mature LCs were exposed to HIV-1 (JRCSF) or serum-opsonized HIV-1 (JRCSF) harvested, extensively washed, and co-cultured with U87 cells. Transmission was determined by antibody staining against p24 and analyzed by flow cytometry. **b**, **c** Epidermal sheets were incubated with CR3 or CR4 antibody and subsequently exposed to HIV-1 (JRCSF or YU2B) or serum-opsonized HIV-1 (JRCSF) or semen-opsonized HIV-1 (YU2B) harvested, extensively washed, and co-cultured with U87 cells. Transmission was determined by antibody staining against p24 and analyzed by flow cytometry. Error bars are the mean ± SD of **a** *n* = 3 donors measured in monoplo, duplicate or triplicate, each dot represents one donor. ****p < 0.0001 by ordinary one-way ANOVA, Tukey post hoc test (multiple comparisons). **b** *n* = 3 donors measured in duplicate or triplicate, each dot represents one donor. ***p < 0.001, ****p < 0.0001 by ordinary one-way ANOVA, Tukey post hoc test (multiple comparisons). **c** *n* = 5 donors measured in duplicate, each dot represents one donor. ****p < 0.0001 by ordinary one-way ANOVA, Tukey post hoc test (multiple comparisons).

Mucosal LCs migrate to the lymph nodes to present antigens to T cells^18^. Immature LCs protect against infection, but activation of LCs leads to infection and infected LCs can transmit HIV-1 to T cells leading to virus dissemination^7,22,23^. Therefore, we investigated the ability of LCs to transmit complement-opsonized HIV-1 to target cells. JRCSF HIV-1 was exposed to either medium or NHS and directly used for transmission. Complement opsonization significantly enhanced HIV-1 transmission by mature LCs to target U87 cells (Fig. 4a). Next, we investigated the effect of complement in the ex vivo tissue transmission model. Human skin explants were exposed to untreated or serum-treated HIV-1 and cultured to allow LCs to migrate from the tissue. After 48 h, migrated LCs were collected and co-cultured with target U87 cells to determine HIV-1 transmission. High concentrations of untreated HIV-1 led to low levels of HIV-1 transmission by LCs from different donors ex vivo (Fig. 4b, c). Strikingly, both serum and semen treatment enhanced HIV-1 transmission by LCs from different donors ex vivo, which was abrogated by blocking CR3 or CR4 (Fig. 4b, c). These data strongly indicate that complement opsonization of HIV-1 in semen increases virus dissemination by LCs and underscore the importance of complement receptors in facilitating HIV-1 infection.

## Discussion

The complement system is an important defense against pathogens. We have shown that HIV-1 subverts the complement system to infect the host and we identified complement as an important component in semen to enhance HIV-1 susceptibility. We have used primary isolated LCs that are restrictive to HIV-1 and show that HIV-1 subverts complement from semen for infection of LCs and viral dissemination. Complement in semen rapidly opsonized HIV-1 and complement-opsonized HIV-1 efficiently infected LCs, leading to virus transmission. LCs, under normal conditions, restrict HIV-1 infection but our data strongly suggest that complement opsonization leads to a different internalization pathway via CR3 and CR4 thereby increasing infection and escaping langerin-mediated degradation. Thus, our data indicate that the complement system in the host and recipient can be an important target to prevent HIV-1 infection. Several studies have shown that semen affects the efficiency of HIV-1 transmission^10–12^. Semen contains components such as mucin 6 preventing transmission of HIV-1 to T cells, by blocking DC-SIGN-mediated transfer of HIV-1 from DCs to CD4 positive T cells^24,25^, whereas cationic polypeptides in semen inhibit HIV-1 infection^26^. In contrast, semen promotes HIV-1 transmission via HIV-1 capture by spermatozoa through heparan sulfates leading to efficient transmission by DCs^27^. Also, proteins such as SEVI in semen enhance viral infection^10,11,28^. Semen contains intrinsic properties that attract LCs to the site of infection^29^. Strikingly, our data showed that complement in SF rapidly opsonized HIV-1, which facilitates virus dissemination via LCs. HIV-1 contains the C1q-binding domain in its envelope glycoprotein gp41, thereby spontaneously activating the classical complement pathway upon entry into the host^13,30,31^. To prevent complement-mediated lysis upon opsonization, HIV-1 incorporates regulators of complement activation during budding; therefore, only low levels of virus particles are lysed by complement activation^32–34^. Activation of the classical pathway and protection against lysis results in accumulated C3-coated HIV-1 particles in semen, which can be transmitted to mucosal surfaces of the recipient. Indeed, we observed deposition of C3b, iC3b, and C3d fragments on the viral surface when HIV-1 was treated with either SF to a similar extent as treatment with serum. Thus, HIV-1 in semen is opsonized by C3 components. Our data strongly suggest that there is no difference between semen- or serum-opsonized virus with relation to infection and transmission. It has been shown that complement-opsonized HIV-1 binds to CR3 and CR4 present on monocyte-derived DCs^35^ leading to infection of immature DCs by overcoming SAMHD1 restriction^36^. Our data here showed that CR3 and CR4 on LCs also efficiently bind complement-opsonized HIV-1 thereby increasing HIV-1 capture. Complement opsonization of the virus may affect the routing of HIV-1 in LCs^15^. LCs express the C-type lectin receptor langerin^37^, which routes HIV-1 into autophagic pathway leading to virus degradation^20,21^. Notably, complement-opsonized HIV-1 interacted with langerin and langerin restricted complement-opsonized HIV-1 infection of langerin-expressing U87 cells in the absence of CR3 and CR4.

However, LCs express CR3 and CR4 and complement-opsonized HIV-1 interacted primarily with CR3 and CR4 but not langerin. We observed internalization of complement-opsonized HIV-1 into LCs. Moreover, complement opsonization increased both fusion and integration of HIV-1 in LCs in a CR3 and CR4 dependent manner. Thus, our data suggest that CR3 and CR4 binding reroutes HIV-1 from the langerin-degradation pathway into vesicles that allow fusion and subsequent integration of HIV-1 in LCs, future studies are required to investigate the limiting restriction by langerin in the presence of CR3 and CR4 on LCs. It has been shown that complement opsonization can sterically hinder sites on the gp120 protein such as the V3 and V1–V2 regions^38^, which might influence recognition by lectins such as langerin and DC-SIGN to interact with HIV-1. However, ectopic expression of langerin blocked infection of U87 cells with complement-opsonized HIV-1 similar to non-opsonized HIV-1. LCs were able to capture opsonized HIV-1 particles leading to an enhancement of HIV-1 fusion, integration, and subsequent infection of LCs. This process was CR3 and CR4 dependent as blocking these receptors abrogated opsonized HIV-1 infection, suggesting that CR3 and CR4 binding of complement-opsonized HIV-1 reroute HIV-1 into LCs. This might explain why mature LCs are susceptible to complement-opsonized HIV-1 infection. Serum as well as SF contained complement and opsonization of HIV-1 strongly enhanced the ability of LCs to transmit HIV-1 via CR3 and CR4 to target cells in the ex vivo transmission model underscoring a role for complement factors in serum and semen as important enhancers of transmission by LCs. Even though langerin is able to block complement-opsonized HIV-1 infection, our data indicate that the virus bypasses langerin internalization by interacting with CR3 and CR4. Thus, suggesting that immune activation in combination with complement opsonization alters the protective function of LCs, mediating HIV-1 infection and subsequent transmission of HIV-1 to target cells, contributing to viral dissemination in the host. Interestingly, our data suggest that complement-opsonization does not affect the interaction with the CLR langerin. We therefore hypothesize that competition of langerin with CR3/CR4 for complement-opsonized HIV-1 leads to rerouting of HIV-1 and subsequent fusion and integration. Further studies are required to investigate whether CR3/CR4 affect langerin signaling and thereby prevent the induction of autophagy, which degrades HIV-1^21^. Novel therapeutical interventions targeting CR3 and CR4 on mature LCs could be considered to counteract cell mediated dissemination of HIV-1.

## Materials and methods

### Antibodies and reagents

The following antibodies were used (all anti-human): CD207-PE mouse IgG1 (#IM3577) (Beckman Coulter, USA), CD1a-APC mouse IgG1 (BD Biosciences, San Jose, CA, USA), CD1a-APC mouse IgG1 (BD Pharmingen), LEAF purified CD11b-AF488 mouse IgG1 (Biolegend, San Diego, CA, USA), LEAF purified CD11c-PE mouse IgG1 (Biolegend, San Diego, CA, USA) and anti-HIV-1 p24, KC57-RD1-PE mouse IgG1, (Beckman Coulter, USA) IgG1 isotype (0.5 mg/mL) (Thermo Fisher Scientific, USA) and Hoechst 33342 (Cell Signaling Technologies). The following reagents were used: 10E2 (anti-langerin) (20 µg/ml)^20^. Semen pooled-human-donors (#991-04-P), liquid, pooled form ≥3 donors (Lee Biosolutions).

### Plasmids, viruses, and cell lines

U87 cell line was obtained through the NIH AIDS Reagent Program, Division of AIDS, NIAID, NIH: U87 CD4+ CCR5+ cells from H. K. Deng and D. R. Littman^39^. Langerin-expressing U87 cells and langerin (W264R) mutant U87 cells were generated as described before^21^. The following virus stocks were obtained through the NIH AIDS Reagent Program, division of AIDS, NIAID: HIV-1 JR-CSF Virus from Dr. Irvin Chen^40,41^. HIV-1 virus stocks were propagated on PHA-stimulated human PBMCs. Produced HIV-1 viruses were quantified by p24 ELISA (Perkin Elmer Life Sciences) and titrated using the indicator cells TZM-bl (John C. Kappes, Xiaoyun Wu, Birmingham, Alabama, USA and TranzymeInc., the NIH AIDS Reagent Program, division of AIDS, NIAID)^42^. JR-SCF determined titer: 4.57 TCID50. Primary isolate 92BR030 (subtype B/B, R5-tropic) was obtained through the National Institutes of Health AIDS (available through World Health Organization depositories). Virus was propagated in PHA-L and IL-2 stimulated PBMCs. YU2B and R9Bal coupled or not to vpr/mCherry were generated by transfecting HEK293T/17 cells (ATCC ACS-4500) with YU2B/R9Bal and mCherry proviral plasmids using calcium-phosphate-mediated transfection^43,44^.

Supernatant was filtrated through 0.22 µm pore-size filters and concentrated by ultracentrifugation at 20,000 rpm for 90 min at 4 °C. Produced HIV-1 stocks were quantified by p24 ELISA and TCID50.

### HIV-1 opsonization

Incubation of HIV-1 with pooled normal human serum (NHS) or SF mediated covalent deposition of C3 fragments (C3b, iC3b, C3d, C3c) on the viral surfaces. Purified YU2B, 92BR030, and JRCSF (concentration > 1 µg p24/ml) were incubated for 1 h at 37 °C with NHS diluted in DPBS (1:10 ratio). YU2B and 92BR030 were incubated for 1 h at 37 °C with SF diluted in DPBS (1:1 ratio). NHS- or SF-opsonized HIV-1 was washed, ultracentrifugated (14,000 rpm for 90 min at 4 °C) and the opsonization pattern was determined. The amount of complement in semen is lower compared to complement in NHS from plasma. Therefore, to obtain similar opsonization levels of HIV-1 by human semen compared to NHS the ratios of opsonization were adjusted. JRCSF viruses were exposed to either NHS or SF and directly used in in vitro or ex vivo settings to mimic the physiological conditions of opsonization.

### Virus capture assay

Virus opsonization patterns were determined by a virus-capture assay (VCA), as previously described^15^. IgG-coated VCA plates were coated with anti-human C3c- (recognizing C3b, iC3b) and C3d and incubated overnight with differentially opsonized virus preparations (1 ng/p24 per well) at 4 °C and extensively washed with RPMI 1640 medium (Sigma) to remove unbound virus. Virus was lysed (2% Igepal) and binding was quantified by p24 ELISA to confirm the opsonization pattern^45^. Human IgG and mouse IgG antibodies were used as control for background binding.

### Ex vivo model and primary LC isolation

Epidermal sheets were prepared as previously described^20,37^. Briefly, skin-grafts were obtained using a dermatome (Zimmer Biomet, Indiana USA). After incubation with Dispase II (1 U/ml, Roche Diagnostics), epidermal sheets were separated from dermis, washed, cut in 1 cm^2^ and cultured in Iscove’s Modified Dulbecco’s Medium (IMDM, Thermo Fischer Scientific, USA) supplemented with 10% FCS (heat-inactivated for 45 min at 56 °C), gentamycin (20 μg/ml, Thermo Fischer Scientific, USA), penicillin/streptomycin (10 U/ml and 10 μg/ml, respectively; Invitrogen). The ex vivo epidermal sheets were used for multiple experimental setups. In vitro mature LCs were generated as described before^20^. Epidermal sheets were cultured for 72 h, mature LCs were harvested, washed, and cultured in IMDM (Thermo Fischer Scientific, USA) supplemented with 10% FCS (heat-inactivated for 45 min at 56 °C), gentamycin (20 μg/ml, Thermo Fischer Scientific, USA), penicillin/streptomycin (10 U/ml and 10 μg/ml, respectively; Invitrogen) at appropriate concentrations. Immature LC-enriched epidermal single-cell suspensions were generated as described before^20,37^. Briefly, epidermal sheets were incubated in PBS containing DNase I (20 units/ml; Roche Applied Science) and trypsin 0.05% (Beckton Dickinson, USA). Single-cell suspension was layered on Ficoll gradient (Axis-shield) and immature LCs were purified using CD1a microbeads (Miltenyi Biotec, Germany). LCs were routinely 85–98% pure and expressed high levels of langerin and CD1a^7,37^.

### Infection and transmission assays and co-culture

For infection and transmission assays two opsonization methods were used. YU2B and 92BR030 viruses that have been opsonized and pelleted to allow standardization of the method (after pelleting the virus concentrations were measured). JRCSF viruses were exposed to either NHS or SF and directly used in in vitro or ex vivo settings.

Human epidermal sheets were incubated with LEAF Purified anti-human CD11b or CD11c (8 µg/ml), and after 2 h exposed to differently opsonized (NHS or SF) HIV-1 JR-CSF (100 µl/sheet, 900 ng p24/ml,), HIV-1 92BR030 or HIV-1 YU2B (100 µl/sheet, 500 ng p24/mL). After 48 h, epidermal sheets were removed, emigrated LCs were harvested, and extensively washed to remove unbound virus. For infection assays LCs (1.0 × 10^5^ cells/well) were cultured for another 3 days, intracellular stained for CD207, CD1a, and p24 KC57-RD1, and analyzed by flow cytometry using FACS Canto II (BD Biosciences). Data were analyzed using FlowJo vX.0.7 software (TreeStar). For transmission assays LCs (1.0 × 10^5^ cells/well) were co-cultured with U87 cells (1.0 × 10^4^ cells/well) for 3 days. Mature LCs were generated as described before and incubated with LEAF Purified anti-human CD11b or CD11c (8 µg/ml), and after 2 h exposed to differently opsonized HIV-1. LCs were extensively washed and co-cultured with U87 cells (1.0 × 10^4^ cells/well) for 3 days. Adherent U87 cells were extensively washed with PBS to remove non-adherent LCs before fixation. Ex vivo LC and in vitro mature LC transmission were determined by intracellular staining of U87 cells for p24 KC57-RD1 and analyzed by flow cytometry using FACS Canto II (BD Biosciences). Data were analyzed using FlowJo vX.0.7 software (TreeStar).

### HIV-1 capture assay

Mature LCs (1.0 × 10^5^ cells/well) were exposed to HIV-1 or complement-opsonized HIV-1 for 6 h at 37 °C, cells were washed to remove unbound virus and capture was quantified by RETRO-TEK HIV-1 p24 ELISA according to manufacturer instructions (ZeptoMetrix Corporation).

### Viral fusion assay

LCs (1.0 × 10^5^ cells/well) were seeded and infected with 250 ng p24/ml of non-opsonized or opsonized R9Bal/β-lam and VSVg/ β-lam. After 5 h incubation, cells were washed and loaded for 1 h with CCF2-AM substrate solution according to the manufacturer’s instructions (LiveBLAzerTM FRET-B/G Loading Kit with CCF2-AM, LifeTechnologies). Cells were washed and developed for 16 h in CO2-independent medium (Gibco) containing 10% FCS and 2.5 mM probenicid. LCs were fixed with 4% paraformaldehyde and cleavage of CCF2 was determined by flow cytometry (FACS Verse, BD Biosciences) and analyzed with FACS Diva software (BD Biosciences).

### HIV-1 integration Alu-PCR assay

Integrated HIV-1 levels were quantified by a two-step Alu-long terminal repeat (LTR) PCR as previously described^46^. Total cell DNA was isolated with peqGOLD DNA Mini Kit (Quiagen) 5 days after infection. For the first round of real-time PCR, the DNA sequence between HIV-1 LTR and the nearest Alu repeat was amplified using Alu-forward primer (5´-TCCCAGCTACTCGGGAGGCTGAGG-3´) and Alu-reverse primer (5´-CCTGCGTCGAGAGATCTCCTCTG-3´). The Alu-HIV-1 PCR cycling conditions included a denaturation step (98 °C for 10 min), followed by 22 cycles of denaturation (98 °C for 30 s), annealing (60 °C for 30 s), and extension (70 °C for 10 min). The second round was a nested quantitative real-time PCR using first-round PCR products and primers to the aforementioned marker region in combination with a HIV-1-specific primer R/U5-forward primer (5´-GGCTAGCTAGGGAACCCACTGC-3´) and R/U5-reverse primer (5´-CTGCTAGAGATTTTCCACACTGAC-3´) by real-time quantitative PCR. Thermal conditions for R/U5 and full-length HIV-DNA included 10 min at 95 °C, 50 cycles of 95 °C for 15 s, and 60 °C for 30 s. Data were analyzed (BioRad CFX Manager Software). HIV-1 integration was normalized relative to GAPDH and uninfected samples were set as 1 for each donor.

### Confocal microscopy

Mature LCs (1.0 × 10^5^ cells/well) were seeded in CellCarrier Ultra plates (Perkin Elmer) and exposed to R9Bal/mCherry or –GFP and YU2B/mCherry (350 ng p24/ml) for 3 h at 37 °C. Cells were fixed with 4% paraformaldehyde, permeabilized (Permeabilization Wash Buffer, Biolegend) and stained using DAPI, CD207, CD1a, and C3b (ImmunoTools). Samples were measured using Operetta High-Content Imaging System (Perkin Elmer), analyzed by HC/HT screening and quantified using Harmony (Perkin Elmer).

### Statistics

For statistical analysis of data sets an ordinary one-way ANOVA or two-way ANOVA, Tukey post hoc test (multiple comparisons) was performed. Statistical analyses were performed using GraphPad Prism 7 software and significance was set at *p < 0.05, **p < 0.01, ***p < 0.001, and ****p < 0.0001.

### Study approval

Studies using human skin tissue from healthy donors was done in accordance with our institutional guidelines with approval of the Medical Ethics Review Committee of the Amsterdam University Medical Centers, location Academic Medical Center (AMC), Amsterdam, the Netherlands, reference number: W15_089 # 15.0103. All samples were handled anonymously.

## Supplementary information

Supplementary information
