# Supplementary material for: HIV-1 subverts the complement system in semen to enhance viral transmission
Source: Mucosal Immunol. 2021 Feb 10;14(3):743–50. doi: 10.1038/s41385-021-00376-9 (PMC8075950; doi:10.1038/s41385-021-00376-9)
Supplement: Supplementary file 1 — Supplementary information [file 41385_2021_376_MOESM1_ESM.pdf]

1

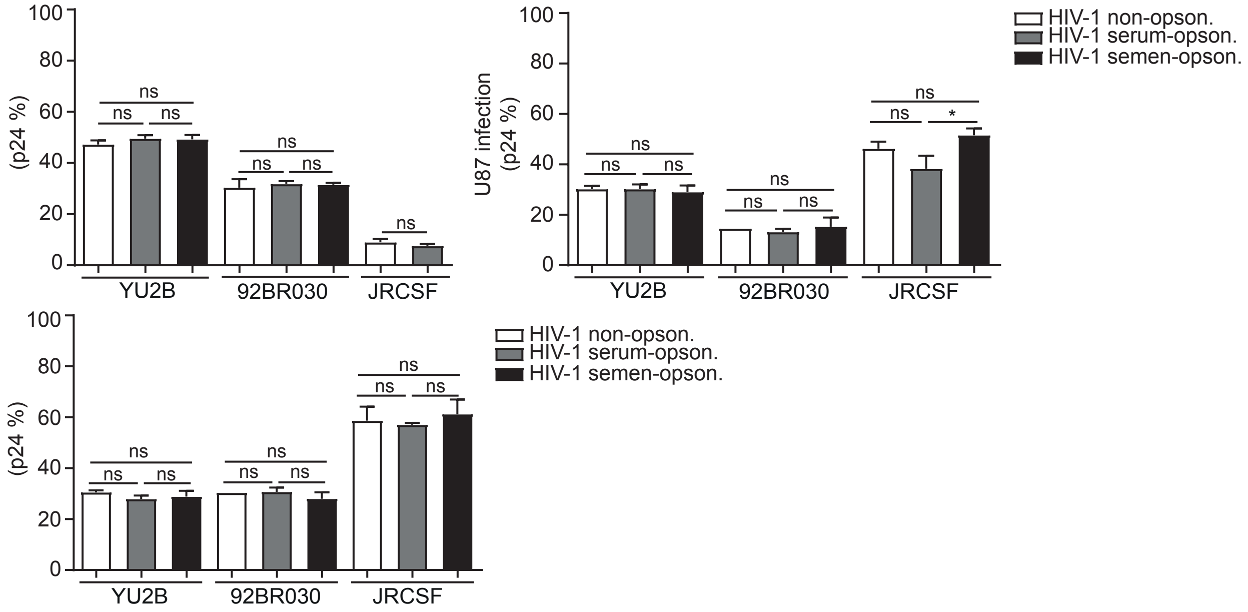


2

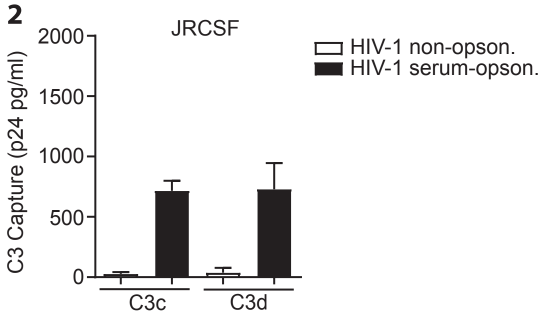


3

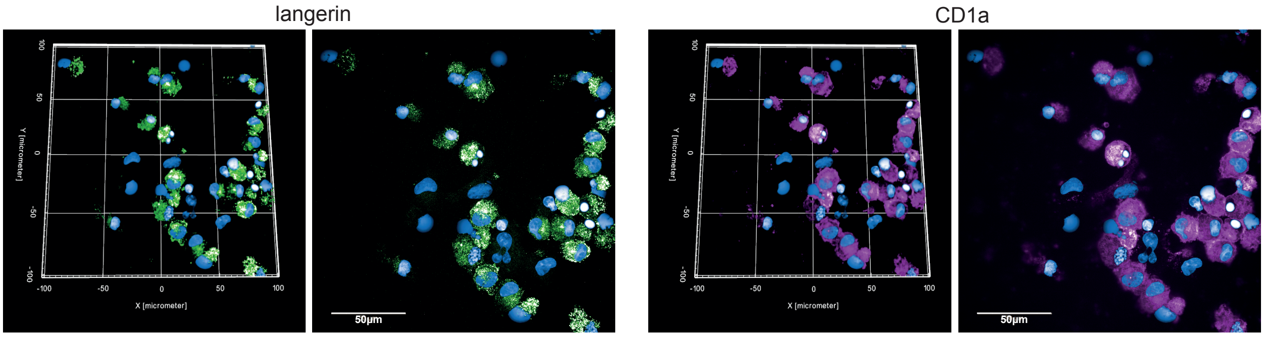


**Supplementary Figures. (S1)** U87 cell line was infected with HIV-1, serum opsonized HIV-1 or semen opsonized HIV-1 for 3 days. Cells were harvested, stained with an antibody against p24 and analysed by flow cytometry. Error bars are the mean ± SD of n=1 experiment measured in duplo. 3 donors are depicted. ns = not significant *(p=0.3684), (p=0.4299), (p=0.9912)* (YU2B), *(p=0.6236), (p=0.7529), (p=0.9733)* (92BR030), *(p=0.6329)* (JRCSF) by Two-way ANOVA, Tukey post hoc test (multiple comparisons). ns = not significant *(p>0.9999), (p=0.9598), (p=0.9630)* (YU2B), *(p=0.4023), (p=0.2133), (p=0.8832)* (92BR030), *(p=0.2168), (p=0.4722),* *p< 0.05 (JRCSF) by Two-way ANOVA, Tukey post hoc test (multiple comparisons). ns = not significant *(p=0.9383), (p=0.9740), (p=0.9918)* (YU2B), *(p=0.1676), (p=0.2741), (p=0.9361)* (92BR030), *(p=0.9755), (p=0.9449) (p=0.8569)* (JRCSF) by Two-way ANOVA, Tukey post hoc test (multiple comparisons). **(S2)** HIV-1 opsonization pattern determined by virus capture assay (VCA) using anti-human C3c- (recognizing C3b, iC3b) C3d. Human IgG was used as control for background binding. Coated VCA plates were incubated overnight at 4°C with JRCSF opsonized virus preparations, washed, bound virus was lysed and binding was quantified by p24 ELISA. Error bars are the mean ± SD of n=3 donors (JRCSF) measured in duplo. **(S3)** Confocal microscopy analysis of mature LCs stained with antibodies against CD207 (A488), CD1a (A647) and nucleus shown in blue (DAPI).

4

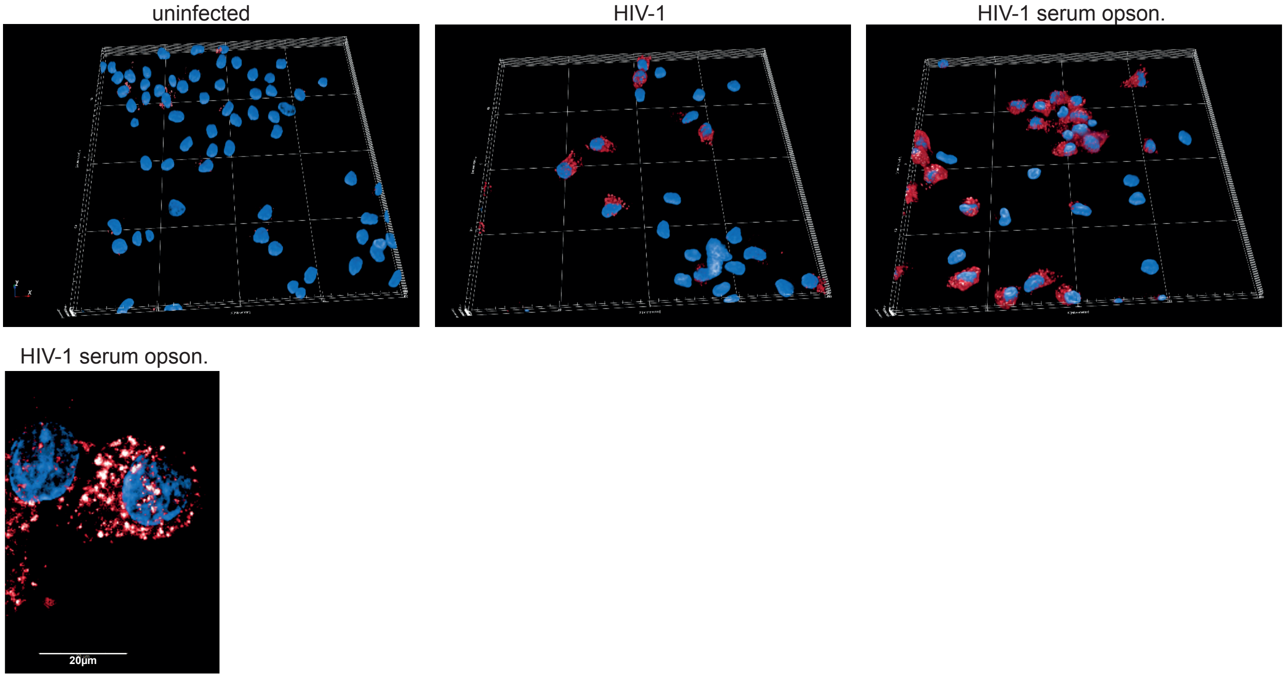


5

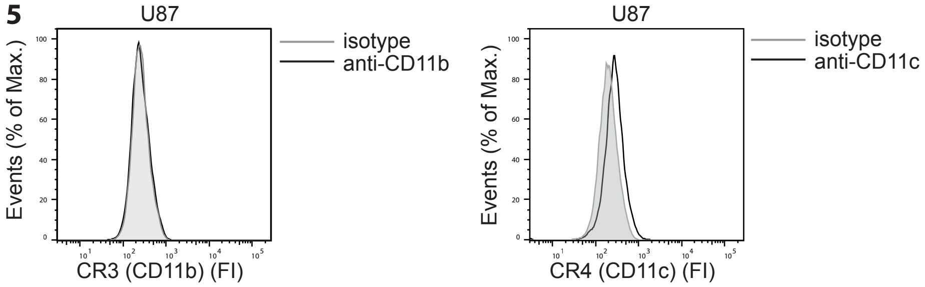


6

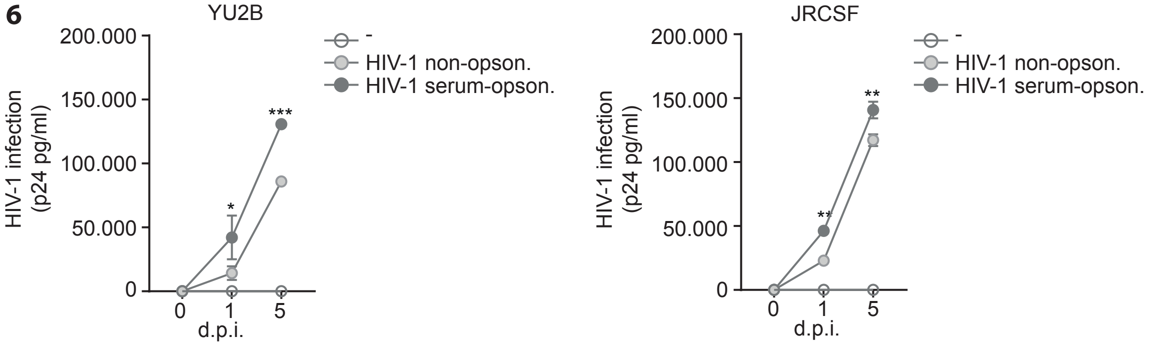


**Supplementary Figures. (S4)** Confocal microscopy analysis of mature LCs exposed to YU2B/mCherry (250ng p24/mL) or serum-opsonized YU2B/mCherry (250ng p24/mL), permeabilized and stained with antibodies against DAPI. HIV-1 and serum-opsonized HIV-1 shown in red (mCherry), nucleus shown in blue (DAPI). Images were taken with a 32x magnification (left) and a 100x magnification for better visualization. Bar = 50 µm and 20 µm. **(S5)** U87 were stained with antibodies against CR3 (CD11b) and CR4 (CD11c) and analyzed by flow cytometry. The histogram shows the cell surface expression of the receptor. One representative experiment out of n=3 is depicted. **(S6)** Mature LCs were infected with 25ng p24/mL of HIV-1 (Yu2B and JRCSF) and serum-opsonized HIV-1 (Yu2B and JRCSF). Supernatant of cultured mature LCs was collected at day 0 (after washing and to remove the unbound virus), day 1 and day 5 post-infection. Productive infection was measured in the supernatant by p24 ELISA. Error bars show mean ± SD from n=2 donors measured in triplicates. *p< 0.05, **p< 0.01, ***p< 0.001 by Two-way ANOVA, Tukey post hoc test (multiple comparisons).

**7**

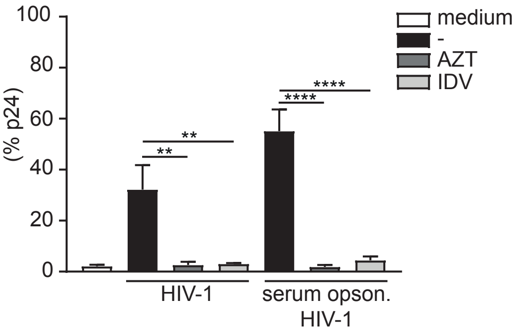


**8**

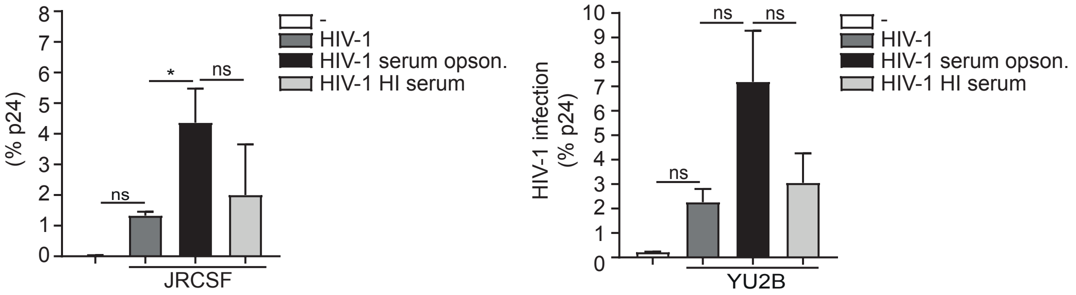


**Supplementary Figures. (S7)** Mature LCs were pre-incubated with AZT or IDV for 2h prior HIV-1 or serum-opsonized HIV-1 infection. Infection was determined by antibody staining against p24 and analyzed by flow cytometry. Error bars are the mean ± SD of n=3 donors measured in duplicates. **p< 0.01, ****p< 0.0001by Two-way ANOVA, Tukey post hoc test (multiple comparisons). **(S8)** Mature LCs were infected with HIV-1 (Yu2B and JRCSF), serum-opsonized HIV-1 (Yu2B and JRCSF) and heat inactivated (HI) serum-opsonized HIV-1 (Yu2B and JRCSF). Infection was determined by antibody staining against CD1a, CD207 and p24 and analyzed by flow cytometry. Error bars are the mean ± SD of n=3 donors measured in duplicates. ns=not significant *(p=0.4789) (p=0.0613),**p< 0.05 (JRCSF), *(p=0.6380), (p=0.1251), (p=0.1968)* (YU2B) by ordinary One-way ANOVA, Tukey post hoc test (multiple comparisons).
